# Doxorubicin, paclitaxel, and cisplatin based chemotherapy for the treatment of angiosarcoma: Two case reports

**DOI:** 10.1016/j.ijscr.2020.02.036

**Published:** 2020-02-21

**Authors:** Joseph A. Lewcun, Colette Pameijer, Rena Kass, Leah Cream, Diane Hershock, Ashton J. Brooks, Daleela G. Dodge

**Affiliations:** Penn State Hershey College of Medicine and Milton S. Hershey Medical Center, Hershey, PA, United States

**Keywords:** Angiosarcoma, Radiation-induced angiosarcoma, Breast cancer, Sarcoma, Neoadjuvant chemotherapy, Case report

## Abstract

**Introduction:**

Angiosarcoma is a rare soft tissue malignancy of endothelial cells, generally associated with a poor prognosis. Due to its rarity, randomized trials are difficult to conduct and a consensus on the treatment of angiosarcoma has not been reached. The role, efficacy, and timing of chemotherapy in AS treatment remain uncertain, and as stated, no large-scale trials have been able to establish definitive recommendations.

**Case descriptions:**

Here we describe the successful use of chemotherapy followed by radiation for a case of lower extremity angiosarcoma, and a case of breast angiosarcoma treated with neoadjuvant chemotherapy followed by surgical resection. Systemic therapy consisted of weekly doxorubicin, paclitaxel, and cisplatin. This regimen resulted in a full clinical remission in the first patient and a pathologic complete response in the second.

**Discussion:**

These cases suggest that the use of the doxorubicin, cisplatin and paclitaxel combination could be an effective alternative to radical surgical excision in extremity sarcomas, and an effective adjuvant treatment to mastectomy in cutaneous radiation-associated angiosarcoma of the breast due to their independent efficacy against angiosarcoma. A randomized trial utilizing neoadjuvant combined doxorubicin, paclitaxel and cisplatin followed by either surgery or radiation, with endpoints assessing pathologic and overall response as well as progression free survival is warranted based on these cases.

**Conclusion:**

The role of neoadjuvant chemotherapy in the treatment of angiosarcoma should be reconsidered considering its ability to provide important prognostic information and improve the likelihood of curative surgery.

## Highlights

• The role and efficacy of chemotherapy in angiosarcoma treatment remains uncertain.
• Doxorubicin, paclitaxel, and cisplatin may be an effective alternative to surgery.
• Chemotherapy may limit the need for debilitating, radical excision in extremity.
• Neoadjuvant chemotherapy may increase chances of curative surgery in the breast.

## 1 Introduction

Angiosarcoma (AS), a malignant proliferation of vascular or lymphatic endothelial cells, develops in approximately 1 per million patients and comprises only 2 % of all soft tissue sarcomas [1–3]. It can occur in any soft-tissue structure, with approximately 15 % developing in the extremity and 20 % developing in the breast [2]. AS can be further subcategorized into those which arise de novo, such as primary breast or soft tissue AS, and those which arise due to secondary factors. Secondary angiosarcoma has a long-known association with both prior radiation therapy (RT) and chronic lymphedema, also referred to as Stewart-Treves syndrome. The incidence of radiation-associated AS of the breast has been estimated as less than one percent amongst patients receiving RT as part of breast conserving treatment [18]. Chronic lymphedema has been shown to be an even more rarely associated risk factor for the development of AS [21].

The overall survival observed across all types of AS is approximately 40 % [25]. Despite the increasingly favorable prognosis observed in other cancers of the breast over recent decades, the 5-year overall survival in AS is still estimated to be 40–50 % [4,5]. As AS is a rare tumor, randomized clinical trials are commonly not feasible and a consensus on treatment guidelines for AS has not been established. Surgical intervention remains the mainstay of management; local wide excision or amputation is often required for AS of the extremity, while mastectomy is often indicated in AS of the breast. For those with AS of the breast, 68 % of patients receive mastectomy or partial mastectomy alone. An estimated 17 % of breast AS patients receive adjuvant radiation therapy and only 6 % receive chemotherapy [5]. The role, efficacy, and timing of chemotherapy in AS treatment remain uncertain, and as stated, no large-scale trials have been able to establish definitive recommendations. Here we present two cases which were successfully treated with a weekly doxorubicin, paclitaxel, and cisplatin-based regimen. The success of the regimen in a case of lower extremity AS led to its subsequent successful use as neoadjuvant therapy in AS of the breast. These cases are reported in accordance with SCARE criteria [26].

## 2 Case report 1

A 59-year-old woman with type 2 diabetes mellitus and long-standing lower extremity edema was referred to our institution for treatment of a T2aN0M1 AS of the left lower extremity. Biopsy at an outside institution demonstrated neoplastic cells with strong diffuse expression of CD31 and pleomorphic spindle cell proliferation within the deep dermis. There was extensive cutaneous involvement with large purple bullae which extended from the medial malleolus to the anterior medial aspect of the knee. MRI imaging showed skin thickening with edema with focal areas of infiltrative abnormal signal and nodular enhancement isolated to the subcutaneous fat.

**Fig. 1:**
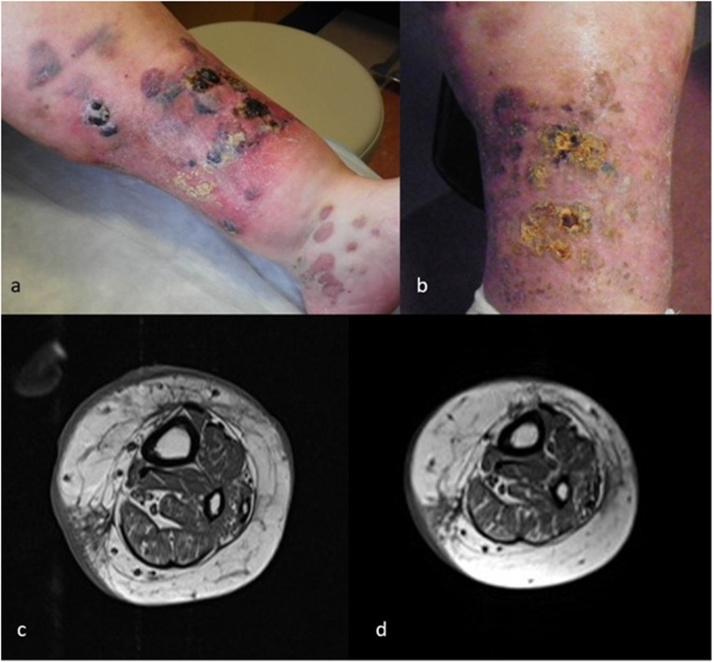
(a) AS of the left lower extremity at first presentation. (b) AS of the left lower extremity after chemotherapy. (c) MRI of left lower extremity angiosarcoma at first presentation. (d) MRI after chemotherapy.

Due to the extent of the malignancy, definitive surgical intervention for curative intent would have required amputation, which would have impeded quality of life and was declined by the patient. Radiotherapy was not practical due to the extent of disease, the large radiation field, and underlying vascular insufficiency. Systemic chemotherapy, which consisted of weekly doxorubicin 20 mg/m^2^ given as a continuous infusion for 18 h on day 1; cisplatin 30 mg/m^2^ on day 2 and paclitaxel 100 mg/m^2^ IV over 3 h on day 2 was selected as the initial treatment. This was given weekly for 3 weeks, every 28 days for 3 cycles. The patient experienced mild side effects which included generalized fatigue and nausea, as well as an episode of acute kidney injury after cycle 1. Carboplatin was substituted for cisplatin during cycle two because of renal insufficiency. However, cisplatin was again given during cycle 3. Six months after diagnosis, repeat MRI showed marked improvement after chemotherapy with decreased skin thickening and edema, and resolution of the deep enhancing nodules. After a disease-free interval of seven months, two new nodules appeared on the medial and lateral aspects of her left foot. Biopsy confirmed localized AS recurrence. The lesions were treated with radiation therapy totaling 30 fractions and 90 Grays. Six, 21-day cycles of single agent paclitaxel 80 mg/m^2^ on days 1, 8 and 15 were added for systemic anti-angiogenic therapy after the completion of radiation therapy. The patient developed peripheral neuropathy as a result of the paclitaxel regimen but otherwise tolerated the treatment well. Screening MRI has been conducted every three months which revealed no concerning findings. There was no evidence of subsequent clinical or radiographic recurrence three years after her diagnosis (Fig. 1).

## 3 Case report 2

A 64-year-old Caucasian female with type 2 diabetes mellitus presented to her breast surgeon with new areas of patchy erythema and ecchymosis on the inferior, medial quadrant of her left breast which had been present for several days. Her medical history was significant for bilateral breast cancer six years prior: a grade II, HER2-positive, ER/PR negative breast cancer in the left breast and a grade III ER/PR positive ductal carcinoma in situ in the right breast. Treatment had included bilateral partial mastectomies performed through reduction mammoplasties, adjuvant chemotherapy, and sequential whole breast radiation therapy. A breast cancer surveillance visit only one month revealed no significant findings on either mammography or clinical breast exam. On physical exam, the erythematous changes were irregularly margined, associated with both a thickening of the overlying skin and two dark purple, well circumscribed bullae-like areas within the confines of a tattoo. Ultrasound of the area revealed no collection or underlying mass. A punch biopsy was performed, and immunohistochemistry demonstrated positive staining for CD31, CD34, factor VIII, and vimentin, supporting the diagnosis of breast angiosarcoma. During the next week, additional lesions appeared involving the medial right breast. An MRI of the breasts showed avidly enhancing skin thickening and nodularity bilaterally, measuring 8 mm in thickness in the medial left breast and 4 mm in the medial, lower quadrant of the right breast.

Due to the bilateral presentation of the angiosarcoma, disease extent within medial locations, and the rapidity of its growth, removal of involved skin by surgical resection would require bilateral mastectomies including en bloc resection of a wide area of skin over the sternum. Primary closure of the defect would not be possible, and the magnitude of the procedure would place the patient at significant risk of post-operative complications and poor healing, delaying any adjuvant treatment. After consultations and presentation at multidisciplinary tumor board, neoadjuvant chemotherapy with the same agents chosen for case report 1 was elected. Two cycles of neoadjuvant chemotherapy consisted of IV doxorubicin 20 mg/m^2^ given over 18 h weekly for three weeks on days 1, 8, and 15, IV paclitaxel 100 mg/m^2^ and IV cisplatin 30 mg/m^2^ infused weekly for three weeks on days 2, 9 and 16. Her chemotherapeutic course was complicated by neutropenia requiring one dose of doxorubicin to be withheld, as well as myalgias, nausea and anemia.

**Fig. 2:**
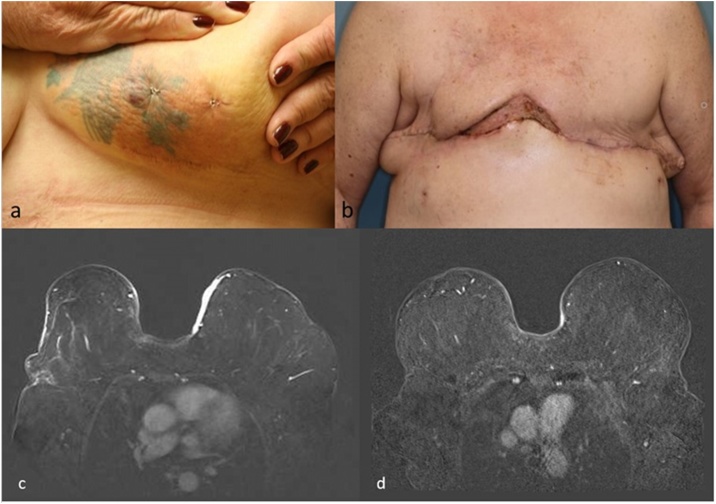
(a) AS of the left, medial breast, prior to chemotherapy. (b) Post bilateral, in-continuity mastectomies with resection of the suprasternal tissue. (c) MRI of bilateral breast AS at first presentation. (d) MRI after neoadjuvant chemotherapy.

All skin changes of the left and right breasts resolved, and a post-treatment MRI demonstrated only minimal residual enhancement. The patient then underwent bilateral, in -continuity mastectomies and resection of the suprasternal tissue with VAC closure of the central defect. Histopathology revealed no residual cancer – a pathologic complete response. The initial plan was for delayed flap closure, however, the area contracted down to a small defect. Satisfactory closure was achieved with a split-thickness skin graft. The patient remains recurrence free two years from the date of diagnosis (Fig. 2).

## 4 Discussion

Angiosarcoma is an aggressive, heterogeneous disease for which there is a lack of consensus on treatment, making prognosis difficult to predict. AS has historically been viewed as a primarily surgical disease, and resection with negative margins remains the standard of treatment [6]. Adjuvant radiotherapy has been shown to moderately improve the recurrence free interval [7]. Only small, retrospective studies have evaluated the benefit of adjuvant chemotherapy, with any conclusions limited by small sample size. Meta-analysis of these studies has demonstrated no decrease in AS recurrence with chemotherapy, though a few studies have reported a benefit after surgical resection with negative margins [8]. Current National Comprehensive Cancer Network (NCCN) guidelines recommend consideration of preoperative chemotherapy only for advanced (stage IIIA-B) disease, which is based on low-level evidence [22]. These two cases demonstrate dramatic, successful treatment with a novel chemotherapy regimen, which was used as definitive treatment in case 1 and as neoadjuvant treatment in case 2.

The first line chemotherapeutic treatments for angiosarcoma have been standard sarcoma anthracycline-based regimens, combined with alkylating agents such as ifosfamide in the metastatic setting. Angiosarcoma sensitivity to anthracyclines, such as doxorubicin, has been shown to be similar to that of other soft tissue sarcomas, with 25 % of AS patients showing a complete or partial response [9]. However, unlike other soft tissue sarcomas, AS has also been shown to be highly sensitive to taxanes. Paclitaxel has been shown to be particularly effective in the treatment of advanced angiosarcoma [10,11]. The role of platinum-based alkylating agents such as cisplatin has not been established. It has been used off-label, in conjunction with doxorubicin for both sarcomas of the bone and soft tissue and was shown to be more effective than single agent doxorubicin in these tumors [12]. A doxorubicin, paclitaxel and cisplatin regimen has been previously reported to produce a long-term remission of metastatic angiosarcoma [13]. Doxorubicin has a relatively small therapeutic index, with potentially severe side effects including cardiotoxicity and myelosuppression [14]. Paclitaxel has been shown to produce cardiac disturbances such as transient asymptomatic bradycardia, as well as neutropenia, neuropathy and paresthesia [15]. However, the cardiac toxicity of doxorubicin and paclitaxel used as combination therapy has been shown to be similar to that of doxorubicin as single agent therapy [16]. Likewise, the extent of myelosuppression observed with doxorubicin and cisplatin combination therapy is nearly equivalent to that of doxorubicin alone [12]. Due to the expression of vascular endothelial growth factor (VEGFR) in AS, the VEGFR/VEGF pathway has also been proposed as a potential therapeutic target. However, clinical trials have shown limited survival benefit of VEGFR/VEGF targeted therapies when compared to current first line chemotherapeutic agents [23,24].

Only a small subset of studies has analyzed the effectiveness of neoadjuvant chemotherapy in the AS population. One study of 15 cases of periorbital angiosarcoma suggested a promising survival benefit [17]. However, the quality and quantity of overall evidence is lacking, and no large-scale trials have been conducted. Both cases illustrated here were in patients with unresectable disease at diagnosis, which necessitated the use of chemotherapy as initial therapy. For the patient in case 1, the extent of disease would have necessitated the loss of the limb and too large a field for radiation due to the risk of significant toxicities. The extent of disease in case 2 was also too widespread for surgical intervention alone. The regimen of weekly doxorubicin, paclitaxel, and cisplatin produced a clinical complete response in both patients. AS is a rapidly growing malignancy and positive margins on resection are common. Thus, a systemic treatment that increases the probability of margin negative resection warrants further study. In a recent single institution review (Brigham and Women’s Hospital, Dan-Farber Cancer Institute) of 33 patients with cutaneous radiation-associated angiosarcoma of the breast, resection of all irradiated skin trended toward better local recurrence-free survival (LRFS) and recurrence-free survival (RFS) [19]. A dramatic reduction in size makes resection far more attainable and further ensures negative margins, which has been proven to be one of the few factors that improves outcome [20]. Neoadjuvant chemotherapy provides important prognostic information and can improve the likelihood of curative surgery. Our first case suggests that the use of the doxorubicin, cisplatin and paclitaxel combination could be an effective alternative to radical surgical excision in extremity sarcomas and an effective adjuvant treatment to mastectomy in cutaneous radiation-associated angiosarcoma of the breast due to their independent efficacy against AS.

Unfortunately, due to the rarity of AS and the diversity of tumor sites, a randomized trial utilizing doxorubicin, paclitaxel and cisplatin to assess overall response and progression free survival is not feasible. Therefore, an alternative means must be considered to generate additional evidence regarding the role of chemotherapy in AS and specifically this regimen’s efficacy. In recent years, cancer databases such as the National Cancer Database (NCDB) and the Surveillance, Epidemiology, and End Results (SEER) program have greatly facilitated the analysis of treatment efficacy and patient outcomes. While these databases provide some data on AS treatment, they fail to report what chemotherapeutic agents were utilized or in what combination. In a number of other rare malignancies, such as adrenal cortical carcinoma and pleuropulmonary blastoma, disease-specific databases have been successfully implemented and have provided extensive insight into tumor characteristics and molecular targets [27,28]. We advocate for the implementation of a similar, disease-specific, national registry of all patients diagnosed with AS. This would allow for a thorough evaluation of treatment methods and create a better understanding of the tumor’s response to specific treatment regimens, including the chemotherapy regimen utilized in our cases.

## Sources of funding

This research did not receive any specific grant from funding agencies in the public, commercial, or not-for-profit sectors.

## Ethical approval

This study was exempt from ethical approval at our institution.

## Consent

Written consent was obtained from the patient for publication of these case reports and accompanying images. A copy of the written consent is available for review by the Editor-in-Chief of this journal on request. Identifying details have been omitted wherever possible.

## Author contribution

**Joseph A. Lewcun:** methodology, investigation, visualization, data curation, writing – original draft, writing – review & editing

**Colette Pameijer:** conceptualization, supervision, writing – review & editing

**Rena Kass:** conceptualization, investigation, writing – review & editing

**Leah Cream:** investigation, validation, writing – review & editing

**Diane Hershock:** investigation, validation, writing – review & editing

**Ashton** J. **Brooks:** conceptualization, investigation, writing – review & editing

**Daleela G. Dodge: guarantor,** conceptualization, supervision, project administration, writing – review & editing

## Registration of research studies

These case reports do not meet the Research Registry’s criteria of “first used in man” as all of these described chemotherapeutic agents have been utilized previously in other settings and for other purposes.

## Guarantor

Daleela G. Dodge.

## Provenance and peer review

Editorially reviewed, not externally peer-reviewed.

## Declaration of Competing Interest

The authors have no conflicting financial or personal interests to disclose.
